# Descriptive and Quasi-Experimental Studies about Moral Emotions, Online Empathy, Anger Management, and Their Relations with Key Competencies in Primary Education

**DOI:** 10.3390/ijerph182111584

**Published:** 2021-11-04

**Authors:** Antonio L. González-Gómez, David P. Farrington, Vicente J. Llorent

**Affiliations:** 1 Department of Education, University of Cordoba, C/San Alberto Magno, s/n., 14004 Córdoba, Spain; m72gogoa@uco.es; 2 Institute of Criminology, Cambridge University, Sidgwick Avenue, Cambridge CB3 9DA, UK; dpf1@cam.ac.uk

**Keywords:** moral emotions, online empathy, anger management, cooperative learning, social and emotional competencies, literacy competence, project-based learning, curricular intervention

## Abstract

Background: Antisocial behaviours make social interactions difficult among students. Moral emotions, online empathy, and anger management are social and emotional variables related to prosocial and antisocial behaviours and health problems. This research aims to assess the impact of Cooperative Project-Based Learning intervention on these three variables for Primary Education students. Additionally, the relations of these variables with key competencies, such as social and emotional competencies and literacy competence, were studied. Method: This research is made up of two studies, descriptive and quasi-experimental, during regular school hours. The descriptive study was carried out with a sample of 516 primary school students and aimed to assess the development of the three variables, taking into account personal and ethnic-cultural factors. The quasi-experimental study, with pre-test and post-test data, had the participation of 145 students to study the incidence of these variables after Cooperative Project-Based Learning intervention in Primary Education. Results: The results show the relation among the cited variables and the positive impact of the intervention on moral emotions and anger management in the experimental group compared to the control group. Experimental group girls presented higher scores in moral emotions than control group girls. Conclusion: These results open new research lines in relation to the intervention as a programme to prevent the appearance of antisocial behaviours and health problems at school.

## 1. Introduction

Positive social relationships can be altered by the appearance of antisocial behaviours, such as aggression, violence, or truancy, among others, which transgress moral and social norms [1]. Antisocial behaviours have been related to moral emotions [2]. Moral emotions are the emotional responses to behaviours related to moral values that promote moral actions [3]. Moral emotions are learned during the relationships that people have in moral events [4]. Some moral emotions are guilt, pride, shame, or satisfaction [5]. These moral emotions increase desirable behaviour [6] and promote moral actions [7]. Guilt feelings have been negatively associated with aggression [8], and higher levels of moral reasoning were positively related to prosocial behaviours in children aged 4 to 5 years [9]. A study [10] identified that six-year-old students, who were classified as prosocial by their teachers, presented moral attributions more frequently. Other research [11] revealed that children’s age was a significant predictor of their aggression, and that the moral issues of the children (4–6 years old) predicted an additional significant portion of the variation in aggressive behaviour. However, more studies are required to analyse the development of moral emotions in Primary Education students.

The appearance of antisocial behaviours is also associated with a low level of empathy [12]. Empathy is a key interpersonal variable present in interpersonal interactions [13] and is defined as “understanding and sharing another’s emotional state or context” [14] (p. 988). This variable is made up of two dimensions [15]; affective empathy (the ability to experience and share the emotional states and emotional contexts of other people) and cognitive empathy (the ability to understand the emotions that other people feel in their emotional context). Empathy is positively related to moral reasoning [16], social awareness, and prosocial behaviour, which are dimensions of social and emotional competencies [17]. Empathy has been studied in Primary Education students [18], where results are contradictory by sex, and in Secondary Education [19], where girls show a higher score than boys.

Prosocial behaviours happen face-to-face, but they also happen when students interact online through social networking sites, online video games, e-mail, chats, or text messages. The adolescent population could misuse both the Internet and online social networks [20,21], which can lead to inappropriate problem behaviours online, such as cyberbullying [22]. Some studies have indicated that low empathy is one of the predictors of cyberbullying [23], finding associations between low levels of empathy and cyberperpetration [24]. Virtual or online empathy [25] is shown to be consistent with interpersonal interactions, and positive and significant correlations have been found between face-to-face and virtual empathy, with lower levels of online empathy than levels of empathy offline. Online interaction may reduce an individual’s empathic capacity because the online connection reduces the amount of time spent interacting face-to-face with others [26]. This interaction can result in a person missing non-verbal communication elements that are essential for reading emotions, such as facial expressions, body posture, eye contact, or gestures. However, online behaviours could support or even increase empathy [27]. Other studies highlight that online empathy does not seem to be clearly related to cyberbullying [28]. These studies provide a controversial view of online empathy, indicating the need for new studies that provide a more complete view of this subject, especially in Primary Education.

Attention control, such as anger management, is a regulating mechanism that is also important to ensure adequate social interaction and avoid antisocial behaviours [29]. Prosocial behaviour is negatively related to anger in students of five, seven, and nine years old [2]. Anger is a primary emotion that is considered harmful and that manifests itself when an individual cannot obtain a goal or satisfy a need [30]. Anger at school is considered an emotional reaction experienced by students [31] that is related positively to social pressure exerted by others [32] and related negatively to self-esteem and optimism [33]. The regulation of emotional behaviours makes it possible to predict socially appropriate behaviours [34]. Controlling attentional processes is important for managing negative emotions, such as anger, and this overt behaviour seems to predict problems of showing these emotions [35]. Consequently, it is necessary to carry out studies in Primary Education that broaden knowledge on anger management, due to its importance to develop adequate social relationships among students from the beginning of the school system.

Schools must promote different dimensions of learning, including cognitive, social, emotional, and moral competencies, in their goal of promoting a comprehensive education [36]. A European Union recommendation [37] advises the development of key competencies in the school curricula. As one of these key competencies, social, emotional, and moral competencies could protect children from bullying [38], future antisocial behaviours, and delinquency among students [39]. Findings in social and emotional learning programs [40] have shown their positive impact on students’ social and emotional skills, academic performance, behaviours, and attitudes. A meta-analysis with 29 (quasi-)experimental studies [41] also shows the positive effects of introducing social and emotional competencies in early-age students’ curricula. However, despite all these positive advantages of incorporating key competencies into the curriculum, they are not worked on in a systematic way.

An intervention based on Project-Based Learning, as a didactic method, could allow students to enhance key competencies, especially social, emotional, and moral competencies. This method promotes active participation in the learning process through social interactions and knowledge exchange [42]. This type of inquiry-based learning promotes solving authentic problems within real-world practices [43]. Project-Based Learning leads to meaningful learning experiences that involve students in their own learning [44]. Recent research [45] showed that Project-Based Learning about values improved many skills, such as recognition of prejudice or empathy. At the same time, cooperation promotes student interaction, where students would share common homework and goals. Cooperative Learning has benefits as a didactic method with the potential to positively affect the intergroup relations of students [46]. Working in small groups allows students to join forces and share resources to enhance their own learning and that of other team members [47]. The assignment of a role to each member of the cooperative base groups [48] ensures interaction and social skills development, such as leadership, decision-making, trust-building, communication, and skills related to conflict management. An intervention, which combined both didactic methods, was carried out in Primary Education [49], and it promoted social and emotional competencies, where both empathy and literacy competence were included. 

Cooperative Project-Based Learning, as a combined method, could also be an effective way to develop moral emotions, online empathy, and anger management in students in Grades Four, Five, and Six of Primary Education. For this reason, the main objective of the study is to analyse the impact of a specific intervention to enhance moral emotions, online empathy, and anger management. Before the analysis of the interventions’ impact, the three variables will also be analysed, taking into account personal factors such as age, sex, or ethnic-cultural group. Moreover, these personal factors will be analysed through their relationship with each other and with other variables, such as social and emotional competencies, empathy, and literacy competence, which have been described in an earlier study [50].

## 2. Materials and Methods

### 2.1. Participants

Two different samples have participated in this research according to the two studies: descriptive and quasi-experimental (see Table 1). Both samples were chosen by incidental sampling from teachers who were interested in participating in the research.

The descriptive study has a sample of 516 students (50.4% boys and 49.5% girls) from six Primary Education schools in Spain, from Grades 4, 5, and 6. The students were distributed as follows: 226 4th graders (43.8%, *M* = 9.17, *SD* = 0.53), 137 5th graders (26.6%, *M* = 10.07, *SD* = 0.31), and 153 6th graders (29.7%, *M* = 11.05, *SD* = 0.34). The students’ age range was 8 to 12 years old (*M* = 9.97, *SD* = 0.88). The sample was divided into two groups, according to their ethnic-cultural group. The majority group consisted of 429 (83.1%) students, and the minority group consisted of 87 students (16.9%). The ethnic-cultural minority group included the gipsy ethnic group and immigrants: 24 (4.7%) students self-identified as members of the gipsy ethnic group (13 with both parents identifying as gipsy and 11 with at least one gipsy parent), and 63 students were immigrants (12.2% of the sample), including those who do not have Spanish nationality or children who, even though they have the Spanish nationality, have at least one of their parents with a foreign nationality. The rest of the students, not included in the minority group, were part of the majority group. The ethnic-cultural component is very similar to the average in the entire region of Andalusia, where there are approximately half a million people of the gipsy ethnic group (6.2%) and where the immigrant population represents 13.5% of the entire population [51,52].

The quasi-experimental study sample consisted of 145 children (47.6% boys and 52.41% girls), with an age range of 8 to 12 years (*M* = 10.03 years, *SD* = 0.92), enrolled in two lines of Grade 4, 5, and 6 during the 2017/2018 academic year. The control group consisted of 71 children (48.6% boys and 51.4% girls), and the experimental group consisted of 74 children (46.5% boys and 53.5% girls). In the same schools, the control group (49%, *M* = 10.04 years, *SD* = 0.89) included those in Grades 4 (*n* = 24), 5 (*n* = 21) and 6 (*n* = 26), and the experimental group (51%, *M* = 10.01 years, *SD* = 0.96) also included those in Grades 4 (*n* = 27), 5 (*n* = 21) and 6 (*n* = 26).

### 2.2. Design and Procedure

The first and descriptive part of the research has an ex-post facto design carried out through a survey of a convenience sample of students in Grades 4, 5, and 6 of Primary Education in the first quarter of the 2017/2018 academic year. The researchers selected six schools from contacts with different teachers who were interested in the scientific study. The survey was anonymous, and the students could refuse completion; it was not completed by 31 students. This research has published its first results about social and emotional competencies, including empathy and literacy competence [18,50]. The data was used to perform correlation analyses with all of the variables of the current study and to reach a more complete view of the profile of the Primary Education students.

The second part of the research develops a quasi-experimental study, with a pre-test and post-test, with a control and an experimental group. The pre-test and post-test were carried out with the participating students at the beginning and at the end of the 2017/2018 academic year. The pre-test questionnaire (Time 1) was completed for all research participants. At the end of that academic year, the questionnaire was given to both groups again, post-test (Time 2). At the beginning of the academic year, the researchers analysed the lesson plan of both groups. Additionally, the researchers were in contact with the teachers during the whole academic year, supervising the lesson plan in both groups. The intervention was carried out only in the experimental group by the regular teachers in the classroom, during regular lessons. The teachers of the experimental group received a three-hour training session for the adequate implementation of the intervention. The teachers were in permanent contact with the researchers during the intervention to facilitate a common experience in the different groups. In the control group, the intervention was not implemented, and students followed textbook activities according to the grade. The control group did not develop any cooperative activity or project-based methods during the academic year.

In both studies, the students completed the questionnaires individually, as one more class activity, during school hours. Data collection was always carried out by a member of the research team, in the presence of the teachers who were responsible for each group. Teachers did not have access to individual questionnaires at any time. The researchers obtained the necessary authorisations, and the participants were informed of the objective of the research, the process, and the expected duration. Ethical Standards of American Psychological Association’s Ethics Code and the Ethical Committee of University of Córdoba (Cordoba, Spain) were followed for the study.

### 2.3. The Intervention in the Quasi-Experimental Study

The teachers of the experimental group students carried out an intervention of Cooperative Project-Based Learning [49] during the 2017/2018 academic year. The intervention is carried out in the literacy area schedule and structured weekly in two phases. Phase 1 is carried out during four sessions of one hour, and its objective is the curricular learning of the specific contents of the literacy area. In these lessons, the teacher uses explanations based on lectures and completing tasks by the students. Phase 2 is developed during a two-hour session that is dedicated to the realisation of the cooperative project.

The cooperative project has as its main objective to create several stories that teach prosocial values: respect, forgiveness, friendship, generosity, tolerance, effort, and peace. Creating a story is sequenced in four activities. The first activity consists of describing characters; in the second activity, the students have to describe a place; in the third activity, they have to make up the story using the characters and the place from the previous activities; and, finally, in the fourth activity, they have to divide the story into scenes, and they could learn and teach the values using a *kamishibai* (a name that refers to material of Japanese origin that is used to tell stories through illustrations). In a periodical inter-class activity, these groups of students tell the stories to groups of students of lower grades. When a group finishes creating a story, they begin creating another. At the end of the academic year, all of these stories created by the different groups were compiled into a book.

The students of the experimental group were grouped in cooperative base groups of 3–4 students. Each cooperative base group includes four roles: spokesperson, evaluator, evaluators’ assistant (when there were four members in the group), and material manager. These roles rotated when project activity was changed. Individually, each student has to propose a personal objective before each of the activities that made up the project. At the end of the activity, each student carries out a self-reflection exercise to achieve this objective. These reflective objectives deal with attitudes and behaviours that the students had with the rest of the partners in the cooperative base group during the interaction of the activities.

The control group students maintained the usual curriculum and did not carry out any similar intervention activities.

### 2.4. Instruments

Information has been collected through an instrument composed of seven parts: personal data (age, sex, nationality, and nationality of the parents and whether or not they belong to the gipsy ethnic-cultural group), *Moral Emotions Scale*, *Online Empathy Questionnaire*, *Anger Management Questionnaire*, *Social and Emotional Competencies Questionnaire*, *Basic Empathy Scale*, and *Literacy Proficiency Test*.

*The Moral Emotions Scale* [53] is made up of 5 items that present Likert type responses from 1 to 5, where 1 is “Totally disagree” and 5 is “Totally agree”. The reliability of the instrument is adequate for the descriptive study sample (α = 0.75 and Ω = 0.77) and for the quasi-experimental study sample, pre-test (α = 0.68 and Ω = 0.70), and post-test (α = 0.79 and Ω = 0.80).

*The Online Empathy Questionnaire* [54] is an instrument based on the *Basic Empathy Scale* [15], and is used to evaluate *empathy* through electronic devices. The instrument is made up of seven items that are Likert type answers from 1 to 5, where 1 is “Totally disagree” and 5 is “Totally agree”. This scale has two factors: *online affective empathy* and *online cognitive empathy*. The reliability of this research was high, in both the descriptive study (α = 0.80 and Ω = 0.80) and the quasi-experimental study, pre-test (α = 0.83 and Ω = 0.83), and post-test (α = 0.73 and Ω = 0.73).

*The Anger Management Questionnaire* is made up of four Likert type response items from 1 to 5, where 1 is “Totally disagree” and 5 is “Totally agree”. Confirmatory factor analysis was performed with the sample of 516 children and shows that the data obtained good scores in the different fit indices (χ^2^ = 12.4102 df = 2; *p* = 0.002; NFI = 0.99; NNFI = 0.97; CFI = 0.965; RMSEA = 0.102; 90% CI = 0.053 to 0.159). The reliability of the instrument is adequate (α = 0.76 and Ω = 0.76) for the descriptive study sample and for the quasi-experimental study sample, pre-test (α = 0.72 and Ω = 0.73), and post-test (α = 0.77 and Ω = 0.78).

*The Social and Emotional Competencies Questionnaire* (SEC-Q) [17] is composed of 16 items that make up four factors of *social and emotional competencies: self-awareness* (four items), *self-management and motivation* (three items), *social awareness and prosocial behaviour* (six items), and *decision-making* (three items). The items are presented with Likert type responses from 1 to 5, where 1 is “Totally disagree” and 5 is “Totally agree”. The instrument has a high reliability in the descriptive (α = 0.80 and Ω = 0.86) and in the quasi-experimental study, pre-test (α = 0.82 and Ω = 0.82), and post-test (α = 0.85 and Ω = 0.85).

*The Basic Empathy Scale* (BES) [15], validated in Spanish by [23], consists of 20 items and includes two factors: *affective empathy* (11 items) and *cognitive empathy* (nine items). The items are presented on a Likert scale where 1 is “Totally disagree”, and 5 is “Totally agree”. The reliability is high in the descriptive study sample (α = 0.83 and Ω = 0.82); quasi-experimental study, pre-test (α = 0.83 and Ω = 0.84), and post-test (α = 0.81 and Ω = 0.82).

*The Literacy Proficiency Test* [18] is based on the PIRLS tests [55] and assesses *listening, reading,* and *writing*. The listening part begins with the teacher reading a story, and the children answer various questions about the story. In the reading part, children answer written questions about a text that they had previously read. In the third part of the test—writing—students write a story based on an image. These tests are evaluated using two scales: the first scale measured comprehension (*listening* and *reading*), and the second scale measured expression (*writing*). Both scales were divided into six levels, including A1 (the most basic/introductory level), A2, B1, B2, C1, and C2 (mastery of proficiency level) based on the criteria from [56]. For example, *writing* A1 level is “He/she vaguely describes tangible realities that know or see using words”, and C2 level is “He/she rebuilds information and arguments from various sources, and presents them in a coherent and originally summarised way”. This instrument has been used by other researchers with the same samples [18,50]. The reliability is high in the descriptive study (α = 0.93 and Ω = 0.93), pre-test (α = 0.86 and Ω = 0.87), and post-test (α = 0.97 and Ω = 0.97). The reliability was calculated by adding up the score of each of the three factors, because it is a polytomous scale.

### 2.5. Data Analysis

The psychometric properties of the questionnaires were checked. The reliability coefficient was analysed through Cronbach’s alpha and McDonald’s Omega, using Factor 10.5.2. In the case of the Anger Management Scale, due to no previous use of the scales in Primary Education children, confirmatory factor analyses were performed with EQS 6.2, which were adjusted to the robust maximum likelihood method and polychoric correlations (Satorra–Bentler chi-square). The data analyses were carried out using SPSS 25, and the effect size was estimated with the Campbell Collaboration calculator.

Descriptive and comparative analyses were carried out with the first sample. The means of the variables with two groups were compared using the Student’s *t*-test, and Levene’s test was performed to identify the homogeneity of the variances. The groups were compared with Cohen’s *d* with 95% CI, to show the strength of the relationships of *moral emotions*, *online empathy*, and *anger management*.

Correlations were calculated between the main study variables (*moral emotions*, *online empathy*, and *anger management*) and age, *social and emotional competencies*, *empathy*, and *literacy competence* to find out possible relationships. The prediction of literacy competence and its factors, as dependent variables, were analysed using a cross-sectional linear regression (all variables at time 1) and with a longitudinal linear regression (predictor variables at time 1 and literacy competence and its factors at time 2).

The progress of the variables after the intervention was analysed with the second sample. The effect size of between-group differences in the pre-test was calculated to know if there were between-group differences both before and after the intervention. This analysis was calculated using a web-based effect-size calculator (the Campbell Collaboration Calculator). The difference in *moral emotions*, *online empathy*, and *anger management* in the longitudinal analyses of the groups—control and experimental—was studied using the ANOVA test of repeated measures. The Mauchly sphericity test was performed to decide if corrections were necessary in the repeated measures ANOVA test, considering *p* < 0.05 significant. The effect size of the ANOVA test of repeated measures was tested with the partial eta squared.

## 3. Results

### 3.1. Moral Emotions by Age, Sex, and Ethnic-Cultural Group in Primary Education

Age did not show a significant correlation with *moral emotions* (*r* = −0.03, *p* = 0.576). According to sex, no significant differences were found in *moral emotions* (*M_boys_* = 21.90, *SD_boys_* = 3.23 vs. *M_girls_* = 22.94, *SD_girls_* = 2.96, *d* = 0.02, 95% CI = −0.15, 0.20). The scores of the students of the ethnic-cultural majority group did not show significant differences from the students of the ethnic-cultural minority group (*M_maj_* = 22.5, *SD_maj_* = 3.10 vs. *M_min_* = 21.91, *SD_min_* = 3.36, *d* = 0.19, CI 95% = −0.05, 0.43).

### 3.2. Online Empathy by Age, Sex, and Ethnic-Cultural Group in Primary Education

Age did not show a significant correlation with *online empathy* (*r* = −0.05, *p* = 0.049), including its two factors; *online empathy affective* (*r* = −0.05, *p* = 0.31) and *online empathy cognitive* (*r* = −0.03, *p* = 0.47). Scores for *online empathy* (*M_boys_* = 22.21, *SD_boys_* = 5.02 vs. *M_girls_* = 22.26, *SD_girls_* = 4.67, *d* = 0.05, 95% CI = −0.13, 0.24) and their *online affective empathy* factor (*M_boys_* = 8.89, *SD_boys_* = 3.04 vs. *M_girls_* = 8.80, *SD_girls_* = 3.06, *d* = −0.03, 95% CI = −0.20, 0.14) showed no significant differences between boys and girls. However, scores for the *online cognitive empathy* factor showed significant differences between girls and boys (*M_boys_* = 13.27, *SD_boys_* = 3.08 vs. *M_girls_* = 13.39, *SD_girls_* = 2.67, *d* = 0.04, 95% CI = −0.14, 0.22). The scores of the students of the ethnic-cultural majority group did not show significant differences from the students of the ethnic-cultural minority group (*M_maj_* = 22.21, *SD_maj_* = 4.92 vs. *M_min_* = 22.51, *SD_min_* = 4.41, *d* = −0.06, 95% CI = −0.32, 0.19) and its factors: *online affective empathy* (*M_maj_* = 8.92, *SD_maj_* = 3.06 vs. *M_min_* = 8.49, *SD_min_* = 2.90, *d* = 0.15, 95% CI = −0.10, 0.39) and *online cognitive empathy* (*M_maj_* = 13.24, *SD_maj_* = 2.90 vs. *M_min_* = 13.90, *SD_min_* = 2.77, *d* = −0.33, 95% CI = −0.49, 0.03).

### 3.3. Anger Management by Age, Sex, and Ethnic-Cultural Group in Primary Education

*Anger management* (*r* = −0.14, *p* = 0.002) had a significant and negative correlation with age. No significant differences were found between boys and girls in *anger management* (*M_boys_* = 12.69, *SD_boys_* = 3.89 vs. *M_girls_* = 14.36, *SD_girls_* = 3.89, *d* = 0.03, 95% CI = −0.15, 0.20). The scores of the students of the ethnic-cultural majority group did not show significant differences from the students of the ethnic-cultural minority group (*M_maj_* = 13.51, *SD_maj_* = 4.07 vs. *M_min_* = 13.48, *SD_min_* = 3.53, *d* = 0.01, CI 95% = −0.23, 0.25).

### 3.4. Correlations between Moral Emotions, Online Empathy, and Anger Management, and Empathy, Social, and Emotional Competencies, and Literacy Competence

*Moral emotions*, *online empathy*, including its *online affective empathy* and *online cognitive empathy* factors, and *anger management* correlated positively and significantly. The correlations also show that *moral emotions* and *anger management* are significantly and positively related to *social and emotional competencies*, including all of its factors, and to *empathy*. *Moral emotions* correlated significantly with both factors: *online affective empathy* and *online cognitive empathy*. However, anger management only correlated significantly and positively with *online cognitive empathy*. *Online empathy* and its two factors correlated significantly and positively with *empathy* and its two factors, and with *social and emotional competencies* and some of its factors. *Moral emotions*, *online empathy,* and *anger management* did not significantly correlate with *literacy competence* (see Table 2).

### 3.5. Moral Emotions, Online Empathy, and Anger Management as Predictors of Literacy Competence

In the descriptive study, a first linear regression was performed to investigate the independent relationships between the predictors and *literacy competence*, controlling for *moral emotions*, *online empathy*, *anger management*, *social and emotional competencies*, *empathy*, and personal variables (age, sex, and ethnic-cultural group membership). Four statistically significant cross-sectional models were estimated (see Table 3): *literacy competence* (R^2^ = 0.32, F = 12.65, *p* < 0.001), *listening* (R^2^ = 0.23, F = 8.59, *p* < 0.001), *reading* (R^2^ = 0.24, F = 8.30, *p* < 0.001), and *writing* (R^2^ = 0.31, F = 11.87, *p* < 0.001). Personal variables (older, being a girl, and ethnic-cultural majority) and self-awareness were predictors in four models. Besides these variables, affective empathy was a predictor of literacy competence, and social-awareness and prosocial behaviour were predictors of listening.

Longitudinal predictors of *literacy competence* were studied through a second linear regression, which was performed with *moral emotions*, *online empathy*, *anger management*, *social and emotional competencies*, *empathy*, and personal variables (age, sex, ethnic-cultural group membership, and intervention group). Four statistically significant longitudinal models were estimated (see Table 4): *literacy competence* (R^2^ = 0.36, F = 3.60, *p* < 0.001), *listening* (R^2^ = 0.39, F = 4.07, *p* < 0.001), *reading* (R^2^ = 0.40, F = 4.19, *p* < 0.001), and *writing* (R^2^ = 0.29, F = 2.54, *p* < 0.01). Intervention group was a predictor in all four models. Besides this variable, age and *self-awareness* were predictors of *literacy competence*, *listening,* and *reading*.

### 3.6. Moral Emotions, Online Empathy, and Anger Management in the Pre- and Post-Tests of the Control and Experimental Groups

No significant differences between pre-test control and experimental groups were found in three variables (see Table 5). However, the post-tests were different; *moral emotions* (F_1,136_ = 6.70, *p* = 0.01, η^2^_p_ = 0.05) and *anger management* (F_1,141_ = 4.10, *p* = 0.045, η^2^_p_ < 0.01) increased significantly after intervention in the experimental group compared to the control group. *Online empathy* (F_1,110_ = 4.38, *p* = 0.59, η^2^_p_ < 0.01) and its *online affective empathy* factor (F_1,126_ = 3.95, *p* = 0.46, η^2^_p_ < 0.01) showed a slight decrease in the scores of both groups, but these were not significant. *Online cognitive empathy* (F_1,115_ < 0.01, *p* = 0.96, η^2^_p_ < 0.01) showed an increase in the scores of the control and experimental groups, but there are also no significant differences.

### 3.7. Change in Moral Emotions, Online Empathy, and Anger Management by Sex

No significant differences at pre-test between control and experimental groups were found in three variables in boys (see Table 6) or girls (see Table 7). The results show that there were no differences between the boys in the control and the experimental groups in any of the variables; *moral emotions* (F_1,62_ = 2.21, *p* = 0.14, η^2^_p_ = 0.03), *online empathy* (F_1,52_ = 0.09, *p* = 0.76, η^2^_p_ < 0.01), nor in their factors; *online affective empathy* (F_1,58_ = 0.23, *p* = 0.64, η^2^_p_ < 0.01) and *online cognitive empathy* (F_1,54_ = 0.17, *p* = 0.68, η^2^_p_ < 0.01); and *anger management* (F_1,65_ = 1.36, *p* = 0.25, η^2^_p_ = 0.02).

As in the boys’ scores, there were no significant differences among the girls in both groups in the *online empathy* scores (F_1,56_ = 0.18, *p* = 0.67, η^2^_p_ < 0.01), including their factors *online affective empathy* (F_1,66_ = 0.35, *p* = 0.56, η^2^_p_ < 0.01) and *online cognitive empathy* (F_1,59_ = 0.17, *p* = 0.69, η^2^_p_ < 0.01), and *anger management* (F_1,74_ = 2.70, *p* = 0.11, η^2^_p_ < 0.01). However, the scores of the girls in the experimental group show significant differences with respect to the scores of the girls in the control group in *moral emotions* (F_1,72_ = 4.39, *p* = 0.04, η^2^_p_ = 0.06).

## 4. Discussion

Moral emotions, online empathy, and anger management are important variables in the social [23], emotional [34], and moral [2] development of students. For this reason, it is necessary to deepen the knowledge of these three variables in Primary Education students. The study’s objective is to assess the impact of an intervention based on Cooperative Project-Based Learning (which adds the development of prosocial values to the regular curriculum of students in Grades Four, Five, and Six of Primary Education) on moral emotions, online empathy, and anger management. Before assessing the impact of the intervention, these three variables were related to age, sex, ethnic-cultural group membership, and variables of educational interest, including two key competencies; social and emotional competencies, including empathy and literacy competence.

Moral emotions have diverse relations with personal characteristics. Moral emotions were not correlated with age. This result would be in line with another study [57], which did not find changes related to age in self-attributed moral emotions of adolescents. In the current study, sex is not related to moral emotions. The results by sex would not be in line with the study results carried out in early childhood [58], where kindergarten girls showed more development of some moral emotions, such as guilt, than boys. Another study [10] also points out that boys in first and second grade are less prosocial in emotional and moral attributions as compared to girls. Probably, the different results among these studies could be explained by the diverse childhood stage; in the current study, the students are in late-Primary Education, in contrast with the early ages of the cited studies. Moral emotions do not show differences by ethnic-cultural group, as related variables such as social and emotional competencies in Primary Education [18]. Thus, the diverse maturation of children could be implicated in moral development. More studies are required to improve learning in the moral area.

There was no relation between online empathy and age. Significant correlation between age and face-to-face empathy were found in 14–25 year olds [19], and children scored higher in cognitive empathy as they grew [59]. The results for online empathy do not show differences between boys and girls. Online empathy has been analysed with face-to-face empathy by sex in adults [25], but this study did not compare differences between boys and girls in online empathy, and boys and girls showed higher scores in face-to-face empathy than online empathy. In the same line, significant relations have been described between face-to-face empathy and online empathy in adults [25], like the results of the current study in Primary Education students. Significant differences in face-to-face empathy were not found between boys and girls in Primary Education [18]. However, other studies on face-to-face empathy in Primary Education [59,60] and adolescence [15,19] showed significant differences between boys and girls, where girls showed significantly higher scores. There were no significant differences in online empathy between ethnic-cultural group membership, in the same way with other studies about face-to-face empathy in Primary Education [18] or in adolescents and early adults [19]. However, low socio-economic status was associated with low face-to-face empathy [15], and associated to a higher face-to-face empathy with a positive family climate [59]. Ethnic-cultural group membership could be associated with socio-economic status or family climate. The inconsistency of these results obtained in relation to online empathy and its relationship with face-to-face empathy highlights the need to initiate more studies, especially longitudinal studies.

Anger management has shown a significant and negative correlation with age, but no significant differences between boys and girls. Boys were more aggressive than girls, and older students were more aggressive than younger ones [10]. The Integrated Cognitive Antisocial Potential (ICAP) theory [61] indicates that antisocial potential is the key construct for committing antisocial acts. The review of the ICAP theory [62] indicates that, depending on age, different models are specified considering various risk factors (tension, modelling, socialisation, impulsivity, and life events), with the influence of parents or colleagues as a factor depending on children’s age. The manifestation of negative emotions, such as anger, can be different at each age, and depend on various factors. Belonging to an ethnic-cultural group did not show significant differences, and this could be explained by many other personal factors, such as risk factors for violent behaviours. Consequently, anger management can be changeable as well. Anger management requires more studies that deepen knowledge about it.

A previous study [50], with the same sample as the descriptive study, seems to indicate that social and emotional competencies, where empathy is included, are predictive variables for literacy competence. This previous evidence has been complemented by moral emotions, online empathy, and anger management. The correlations show relationships between moral emotions, online empathy, and anger management and social and emotional competencies and face-to-face empathy, but not with literacy competence, and neither with any of its factors. The results of the present study confirm the previous relations among variables [50], even after introducing moral emotions, online empathy, and anger management. The cross-sectional linear regression showed that the self-awareness factor is a predictor of literacy competence, as well as in the longitudinal linear regression. These results support the previous results [50] about the relationships between social and emotional competencies and literacy competence. However, moral emotions, online empathy, and anger management are not predictive variables of the literacy competence level after one academic year, while experimental group membership is a predictor of literacy competence. These results reveal the interconnections among social and emotional variables and the importance of specific lesson plans to develop literacy competence.

The results of the current research show that the use of Cooperative Project-Based Learning has produced benefits in the moral emotions and anger management of the experimental group by including prosocial values in the literacy area. In the same way, this happened in another study [49] with social and emotional competencies and face-to-face empathy. Cooperative learning has a positive impact on intergroup relationships [46]. Cooperative base groups improved social skills [63]. Hence, including cooperative base groups in a Project-Based Learning programme could be a positive way to improve social, emotional, and moral competencies. The inclusion of the self-assessment of behaviour and the inclusion of tasks that explicitly incorporate social, emotional, and moral content and values in the school curriculum could also contribute to the social, emotional, and moral development of students. A cooperative programme in Primary Education [64] improved students’ emotional control and regulation, and empathy. However, online empathy did not show significant differences between the control and experimental groups. This may be because the intervention did not incorporate the use of technological and digital media or applications, which are present issues to consider in future school practice and educational research.

The intervention shows more unexpected results, such as that girls in the experimental group showed significant differences in the scores of moral emotions compared with the girls in the control group, while the boys in the experimental group did not show significant differences from the scores of the boys in the control group in any of the variables. A quasi-experimental study [49] also found this difference by sex in social and emotional competencies and empathy in the pre-test and post-test comparisons of the intervention. Along the same lines, a study of a two-year social and emotional intervention [65] showed that the differences between boys and girls persisted even after the intervention. A longitudinal research study [66] demonstrated that male sex was one of the predictors of future violence. Education could facilitate sex equality and reduce the differences between boys and girls, as suggested by other studies about teacher training [67]; however, a way to generate a significant impact on boys would have to be found.

## 5. Conclusions

The descriptive study shows the development of moral emotions, online empathy, and anger management in Primary Education, detailing analyses by sex and age. This study also showed the existing correlation with social and emotional competencies, including face-to-face empathy. The quasi-experimental study showed the positive impact of a Cooperative Project-Based Learning intervention on moral emotions and anger management when elements of social, emotional, and moral learning are introduced into literacy plan lessons.

Schools must contribute to the total development of students. For this reason, the Council of Europe [56] advises the incorporation of key competencies in national curricula, as a base for the teaching–learning process. Social, emotional, and moral competencies are included in these key and basic competencies, but their incorporation into the school curriculum has difficulties, because they are not associated with any particular subject. The inclusion of social and emotional competencies in the school curriculum, in areas such as language, has been shown to produce benefits for students [68,69]. The intervention in the current study presents added value, since it incorporates social, emotional, and moral competencies in the curriculum, specifically in the literacy subject. The results have implications for school management to guarantee the acquisition of the key competencies in lesson plans.

The present study yields hopeful findings for improving the education of Primary Education students. The sample size is a limitation, which does not allow wide generalisation of the conclusions. Therefore, it is necessary to continue more studies on Primary Education, with more participants involved in other educational stages, to expand the knowledge of the study variables. The findings open new lines of research.

The first line of research would be to analyse the possible causes of personal differences—as well as among different variables—and effects of new proposals that affect social, emotional, and moral development. The differences by sex were shown in the descriptive part of the study. New research is needed to verify didactic and organisational innovations that also allow enhancing the development of the social, emotional, and moral aspects in boys in pursuit of gender equality. Roseth [70] suggests that, if a cooperative base team would be formed by people who were committed to the wellbeing of other partners, the programme would be more successful. It is possible that present results depended on the personal skills of cooperative base team members. The results in general are inconsistent according to sex, and more research is needed, especially including other personal factors. In the case of the current descriptive study, differences are shown by sex. For this reason, social, emotional, and moral development will probably be different. A guideline to improve social, emotional, and moral competencies in Primary Education students could be organised, taking into account the cooperative base groups according to social skills, instead of other personal characteristics, such as sex.

The second research line would provide more information on the relationship between moral emotions, online empathy, anger management, and antisocial behaviours at school. A study carried out in middle schools [71] indicated that the cooperative methodology facilitates the reduction of bullying and violence in the classroom. Other studies [72,73] have shown that the development and promotion of social and emotional competencies is protective against antisocial behaviours and health problems at school. Therefore, it would be interesting and pertinent to continue studying Cooperative Project-Based Learning, including other study variables related to antisocial behaviours, such as bullying or cyberbullying, which are usually connected to health problems. These studies would indicate whether this intervention can be used in the prevention or reduction of antisocial behaviours. Aggression and antisocial behaviours can be considered an expression of social exclusion. Therefore, social and emotional learning integrated into the curriculum of the key competencies can promote an inclusive education for all students. For this reason, it is important to carry out more studies about the development of key competencies through Cooperative Project-Based Learning. The results of future studies could provide evidence to propose didactic innovations that contribute to the greater development of social, emotional, and moral competencies, especially in boys, promoting gender equality and inclusive education.

## Figures and Tables

**Table 1:** Participants.

	Descriptive Study	Quasi-Experimental Study
*N*	516	145
Age	8 to 12 years (*M* = 9.97, *SD* = 0.88)	8 to 12 years (*M* = 10.03 years, *SD* = 0.92)
Boys	50.4% (*n* = 260)	47.6% (*n* = 69)
Girls	49.5% (*n* = 256)	52.4% (*n* = 76)
Grade 4	226 (43.8%, *M* = 9.17, *SD* = 0.53)	Control group (*n* = 24, 16.6%, *M* = 9.04; *SD* = 0.20)
		Experimental group (*n* = 27, 18.6%, *M* = 8.96, *SD* = 0.34)
Grade 5	137 (26.6%, *M* = 10.07, *SD* = 0.31)	Control group (*n* = 21, 14.5%, *M* = 10.05, *SD* = 0.22)
		Experimental group (*n* = 21, 14.5%, *M* = 10.05, *SD* = 0.22)
Grade 6	153 (29.7%, *M* = 11.05, *SD* = 0.34)	Control group (*n* = 26, 17.9%, *M* = 10.96, *SD* = 0.53)
		Experimental group (*n* = 26, 17.9%, *M* = 11.08, *SD* = 0.40)
Ethnic-cultural group	Majority group = 429	There are no ethnic-cultural participants.
	Minority group = 87	

**Table 2:** Correlations among moral emotions, online empathy, anger management, literacy competence, social and emotional competencies, and empathy.

	*Moral Emotions*	*Online Affective Empathy*	*Online Cognitive Empathy*	*Online Empathy*	*Anger Management*
*Moral Emotions*					
*Online Affective Empathy*	0.14 **				
*Online Cognitive Empathy*	0.13 **	0.33 **			
*Online Empathy*	0.17 **	0.83 **	0.81 **		
*Anger management*	0.22 **	0.14 **	0.11 *	0.15 **	
*Self-awareness*	0.22 **	0.06	0.08	0.08	0.28 **
*Self-management and motivation*	0.25 **	0.06	0.10 *	0.11 *	0.22 **
*Social awareness and prosocial behaviour*	0.41 **	0.10 *	0.18 **	0.17 **	0.41 **
*Decision-making*	0.23 **	0.06	0.12 **	0.10 *	0.33 **
*Social and emotional competencies*	0.38 **	0.11*	0.17 **	16 **	0.42 **
*Affective Empathy*	0.25 **	0.34 **	0.13 **	0.30 **	0.07
*Cognitive Empathy*	0.32 **	0.11 *	0.15 **	0.14 **	0.27 **
*Empathy*	0.34 **	0.30 **	0.17 **	0.29 **	0.18 **
*Listening*	0.06	−0.03	−0.04	−0.04	−0.01
*Reading*	0.08	0.01	−0.05	−0.03	0.02
*Writing*	0.09 *	0.01	−0.04	−0.02	−0.02
*Literacy competence*	0.09	<0.01	−0.05	−0.03	<0.01

* Correlations are significant at the 0.01 level (2-tailed). ** Correlations are significant at the 0.05 level (2-tailed).

**Table 3:** Linear regression analyses with *moral emotions*, *online empathy*, *anger management*, *socio-emotional competencies*, *empathy,* and personal variables (age, gender, and ethnic-cultural group membership) as cross-sectional predictors of *literacy competence* (*n* = 516).

	*Literacy Competence*		*Listening*		*Reading*		*Writing*	
	*β*	*t*	*β*	*t*	*β*	*t*	*Β*	*t*
*Moral emotions*	−0.00	−0.33	−0.00	−0.17	−0.00	−0.38	−0.00	−0.09
*Online affective empathy*	−0.00	0.26	−0.00	−0.35	0.01	0.83	0.00	0.05
*Online cognitive empathy*	−0.01	−1.03	−0.00	−0.23	−0.01	−1.01	−0.01	−0.96
*Anger management*	−0.00	−0.43	−0.00	−0.35	0.00	0.32	−0.01	−1.04
*Self-awareness*	0.03 *	2.82	0.03 **	2.67	0.02 *	2.14	0.03 *	2.31
*Self-management and motivation*	−0.01	−0.94	−0.02	−1.33	−0.02	−1.42	0.00	0.96
*Social-awareness and prosocial behaviour*	0.02	1.80	−0.02 *	2.22	0.02	1.58	0.02	1.06
*Decision-making*	−0.07	−0.56	0.00	−1.42	−0.00	−0.32	0.00	0.30
*Affective empathy*	0.01 *	2.13	0.00	1.17	0.01	1.68	0.01 *	2.32
*Cognitive Affective*	0.00	0.13	0.00	0.16	−0.00	−0.18	0.00	0.19
Age	0.26 **	9.79	0.20 **	7.31	0.22 **	7.79	0.36 **	9.80
Sex	−0.14 *	−2.88	−0.14 **	−2.74	−0.14 *	−2.68	−0.16 *	−2.29
Ethnic-cultural group	−0.27 **	−3.85	−0.23 **	−3.18	−0.23 *	−3.12	−0.37 **	−3.47

* *p* < 0.05; ** *p* < 0.01.

**Table 4:** Linear regression analyses with *moral emotions*, *online empathy*, *anger management*, *socio-emotional competencies*, *empathy*, and personal variables (age, gender, ethnic-cultural group membership, and intervention group) as longitudinal predictors of *literacy competence* (*n* = 145).

	*Literacy Competence*		*Listening*		*Reading*		*Writing*	
	*Β*	*t*	*β*	*t*	*β*	*t*	*Β*	*t*
*Moral emotions*	0.14	0.91	0.05	0.94	0.02	0.49	0.07	1.11
*Online affective empathy*	−0.13	−1.05	−0.04	−1.06	−0.05	−1.29	−0.04	−0.75
*Online cognitive empathy*	−0.16	0.98	−0.06	−1.45	−0.05	−1.18	−0.06	−0.98
*Anger management*	0.04	0.40	0.02	0.63	0.02	0.78	−0.00	−0.01
*Self-awareness*	0.32 *	2.13	0.11 *	2.25	0.10 *	2.19	0.11	1.79
*Self-management and motivation*	−0.09	−0.59	−0.03	−0.60	−0.03	−0.53	−0.04	−0.56
*Social-awareness and prosocial behaviour*	−0.05	−0.38	−0.02	−0.55	−0.02	−0.59	−0.00	−0.05
*Decision-making*	−0.25	−1.75	−0.08	−1.91	−0.08	−1.90	−0.08	−1.34
*Affective empathy*	0.07	1.53	0.02	1.39	0.03	1.94	−0.02	1.17
*Cognitive empahy*	0.08	0.98	0.03	1.01	0.03	0.35	0.03	0.88
Age	1.17 **	3.38	0.44 **	4.18	0.45 **	4.19	0.28	1.89
Sex	−0.55	−0.84	−0.05	−0.22	−0.10	−0.52	−0.40	−1.44
Ethnic-cultural group	−2.68	−1.66	−0.56	−1.12	−0.86	−1.71	−1.27	−1.84
Intervention group	2.56 **	−1.66	0.79 **	4.02	0.82 **	4.18	0.95 **	3.50

* *p* < 0.05; ** *p* < 0.01.

**Table 5:** Differences in pre-and post-test scores in *moral emotions*, *anger management*, and *online empathy* in the control and experimental groups.

	Control (*n* = 71)		Experimental (*n* = 74)				
	Pre-Test *M* (*SD*)	Post-Test *M* (*SD*)	Pre-Test *M* (*SD*)	Post-Test *M* (*SD*)	Differences between the Pre-Tests *d* (IC)	Differences between the Post-Tests *d* (IC)	Differences between the Pre- and Post-Tests F(*p*)
*Moral Emotions*	22.52 (2.38)	21.26 (4.04)	22.63 (2.54)	22.83 (2.36)	0.03 (−0.30, 0.35)	0.48 (0.15, 0.82)	6.70 (0.01) *
*Online Affective Empathy*	8.55 (3.24)	7.61 (2.65)	8.97 (2.82)	8.52 (2.68)	0.14 (−0.19, 0.47)	0.39 (0.04, 0.73)	0.56 (0.46)
*Online Cognitive Empathy*	12.60 (2.54)	12.84 (2.20)	13.13 (2.63)	13.40 (2.65)	0.14 (−0.20, 0.47)	0.12 (−0.23, 0.48)	<0.01 (0.96)
*Online Empathy*	21.26 (4.76)	20.53 (3.85)	22.06 (4.28)	21.88 (4.39)	0.17 (−0.16, 0.51)	0.06 (−0.30, 0.41)	0.29 (0.59)
*Anger management*	13.73 (3.82)	12.67 (4.08)	12.68 (3.99)	13.03 (4.26)	−0.21 (−0.54, 0.011)	0.09 (−0.24, 0.41)	4.10 (0.045) *

* *p* < 0.05.

**Table 6:** Differences in pre-and post-test scores in *moral emotions*, *anger management*, and *online empathy* in boys.

	Control		Experimental			
	Pre-Test *M* (*SD*)	Post-Test *M* (*SD*)	Pre-Test *M* (*SD*)	Post-Test *M* (*SD*)	Differences between the Pre-Tests *d* (IC)	Differences between the Pre- and Post-Tests F(*p*)
*Moral Emotions*	22.22 (2.49)	21.29 (3.48)	21.67 (2.83)	21.97 (2.69)	0.21 (−0.27, 0.68)	2.21 (0.14)
*Online Affective Empathy*	8.94 (3.22)	7.87 (2.83)	9.36 (2.72	8.73 (2.65)	−0.14 (−0.62, 033)	0.23 (0.64)
*Online Cognitive Empathy*	13.10 (2.58)	13.28 (2.37)	13.23 (2.66)	13.64 (2.76)	−0.05 (−0.54, 0.44)	0.17 (68)
*Online Empathy*	22.23 (4.89)	21.21 (4.41)	22.21 (4.48)	22.28 (4.42)	−0.07 (−0.56, 0.42)	0.09 (0.76)
*Anger management*	13.12 (4.02)	12.03 (3.90)	12.87 (4.06)	12.58 (4.22)	0.28 (−0.20, 0.75)	1.36 (0.25)

**Table 7:** Differences in pre-and post-test scores in *moral emotions*, *anger management*, and *online empathy* in girls.

	Control (*n* = 37)		Experimental (*n* = 38)			
	Pre-Test *M* (*SD*)	Post-Test *M* (*SD*)	Pre-Test *M* (*SD*)	Post-Test *M* (*SD*)	Differences between the Pre-Tests *d* (IC)	Differences between the Pre- and Post-Tests F(*p*)
*Moral Emotions*	22.68 (2.29)	21.24 (4.45)	23.34 (2.22)	23.61 (1.70)	−0.30 (−0.75, 0.1594)	4.39 (0.04) *
*Online Affective Empathy*	8.08 (3.19)	7.35 (2.48)	8.44 (2.90)	8.53 (2.93)	−0.12 (−0.58, 0.34)	0.35 (0.56)
*Online Cognitive Empathy*	12.19 (2.49)	12.41 (2.11)	12.68 (2.73)	12.50 (2.77)	−0.19 (−0.65, 0.28)	0.16 (0.69)
*Online Empathy*	20.32 (4.73)	19.83 (3.37)	21.29 (4.50)	20.92 (4.42)	−0.21 (−0.68, 0.26)	0.18 (0.67)
*Anger management*	13.87 (3.95)	12.50 (4.14)	13.29 (4.04)	13.45 (4.31)	0.15 (−0.31, 0.60)	2.70 (0.10)

* *p* < 0.05.

## Data Availability

The data presented in this study are available on request from the corresponding author. The data are not publicly available due to privacy police and data protecttion.
